# Clinical evaluation of a multiplex droplet digital PCR for diagnosing suspected bloodstream infections: a prospective study

**DOI:** 10.3389/fcimb.2024.1489792

**Published:** 2025-01-16

**Authors:** Yaqin Peng, Ruijie Xie, Yifeng Luo, Penghao Guo, Zhongwen Wu, Yili Chen, Pingjuan Liu, Jiankai Deng, Bin Huang, Kang Liao

**Affiliations:** ^1^ Department of Clinical Laboratory, The First Affiliated Hospital, Sun Yat-sen University, Guangzhou, China; ^2^ Division of Pulmonary and Critical Care Medicine, The First Affiliated Hospital, Sun Yat-sen University, Guangzhou, China; ^3^ Institute of Respiratory Diseases, Sun Yat-sen University, Guangzhou, China; ^4^ Department of Emergency, The First Affiliated Hospital, Sun Yat-sen University, Guangzhou, China

**Keywords:** bloodstream infection, blood culture, droplet digital PCR, pathogen load, predictive value

## Abstract

**Background:**

Though droplet digital PCR (ddPCR) has emerged as a promising tool for early pathogen detection in bloodstream infections (BSIs), more studies are needed to support its clinical application widely due to different ddPCR platforms with discrepant diagnostic performance. Additionally, there is still a lack of clinical data to reveal the association between pathogen loads detected by ddPCR and corresponding BSIs.

**Methods:**

In this prospective study, 173 patients with suspected BSIs were enrolled. A multiplex ddPCR assay was used to detect 18 pathogens. The results of ddPCR testing were evaluated in comparison with blood cultures (BCs) and clinical diagnosis. Taking BC as the gold standard, receiver operating characteristic curve and Cohen’s kappa agreement were used to investigate whether the pathogen load could predict a corresponding culture-proven BSI for the top five microorganisms detected by ddPCR.

**Results:**

Of the 173 blood samples collected, BC and ddPCR were positive in 48 (27.7%) and 92 (53.2%) cases, respectively. Compared to BC, the aggregate sensitivity and specificity for ddPCR were 81.3% and 63.2%, respectively. After clinical adjudication, the sensitivity and specificity of ddPCR increased to 88.8% and 86.0%, respectively. There were 143 microorganisms detected by ddPCR. The DNA loads of these microorganisms ranged from 30.0 to 3.2×10^5^ copies/mL (median level: 158.0 copies/mL), 72.7% (104/143) of which were below 1,000 copies/mL. Further, statistical analysis showed the DNA loads of *Escherichia coli* (AUC: 0.954, 95% CI: 0.898-1.000, κ=0.731, cut-off values: 93.0 copies/mL) and *Klebsiella pneumoniae* (AUC: 0.994, 95% CI: 0.986-1.000, κ=0.834, cut-off values: 196.5 copies/mL) were excellent predictors for the corresponding BSIs. The DNA loads of *Pseudomonas aeruginosa* (AUC: 0.816, 95% CI: 0.560-1.000, κ=0.167), *Acinetobacter baumannii* (AUC: 0.728, 95% CI: 0.195-1.000), and *Enterococcus* spp. (AUC: 0.282, 95% CI: 0.000-0.778) had little predictive value for the corresponding culture-proven BSIs.

**Conclusion:**

Our results indicate that the multiplex ddPCR is a promising platform as a complementary add-on to conventional BC. The DNA loads of *E. coli* and *K. pneumoniae* present excellent predictive value for the corresponding BSIs. Further research is needed to explore the predictive potential of ddPCR for other microorganisms.

## 1 Introduction

Bloodstream infection (BSI) is a major public health burden worldwide, often leading to septic shock and death (Lamy et al., 2020; Massart et al., 2021). Rapid and accurate pathogen diagnostics are decisively valuable for the early administration of appropriate antimicrobials for patients with BSIs, which are exceedingly crucial for improving prognosis and decreasing the all-cause mortality rate in BSIs (Timsit et al., 2020).

Until now, blood culture (BC) remains the gold standard and first-line tool for pathogen identification and antimicrobial susceptibilities test (AST) in BSIs (Lamy et al., 2020). Although major improvements have been made in the diagnostic performance of BC, low sensitivity and long turnaround time still plague this technology as it is mostly limited by the amounts and growth rates of circulating microorganisms and the use of antibiotics prior to blood collection (Cheng et al., 2019; Lin et al., 2023).

In the last decade, culture-free molecular technologies have shown great potential in providing early pathogen detection, antibiotic efficacy, and prognosis evaluation in BSIs (Li et al., 2021; Merino et al., 2022; Timsit et al., 2020). Droplet digital polymerase chain reaction (ddPCR) is a third-generation PCR technology, in which the reaction solution is divided into thousands of partitions using emulsified microdroplets suspended in oil, and target copy number is counted using Poisson distribution analyses of positive signals after independent PCR amplification (Pinheiro et al., 2012). Compared to other molecular technologies, ddPCR is thought to be faster and have higher sensitivity and precision in detecting target pathogens in BSIs (Abram et al., 2020; Hu et al., 2021; Zheng et al., 2021). It is also capable of absolutely quantifying target molecules without the need to generate a calibration curve, which is an incomparable advantage (Huggett et al., 2015). However, different ddPCR platforms have variable sensitivities and specificities for diverse microorganisms at various infection sites (Wu et al., 2022). To support a wide clinical application, more data are needed to validate and interpret the discrepant ddPCR results when diagnosing BSIs in clinical practice.

However, these molecular techniques have still been proven suboptimal due to some limitations (Lamy et al., 2020). One of the biggest problems is that it is difficult to distinguish whether the agents detected by these methods are true BSI pathogens or not, particularly for low-level conditional pathogens commonly found in clinical settings. There are no clear cut-offs for differentiating infection from colonization or contaminants. In addition, a high detection rate of multiple pathogens using these molecular methods makes it more difficult, since polymicrobial bacteremia is thought to be relatively rare (Peri et al., 2022; Wu et al., 2022). Though several studies have indicated that ddPCR has a satisfactory performance in the early diagnosis of BSIs (Merino et al., 2022), there is a lack of clinical data to reveal the association between the absolute quantification of a pathogen and the corresponding BSI.

In this prospective study, the performance of ddPCR testing was evaluated carefully based on BC results in patients with suspected BSIs. For the first time, we attempted to explore whether the ddPCR-calculated DNA load of a pathogen could be a potential marker for a corresponding culture-proven BSI.

## 2 Materials and methods

### 2.1 Study design and patients

**Figure 1:**
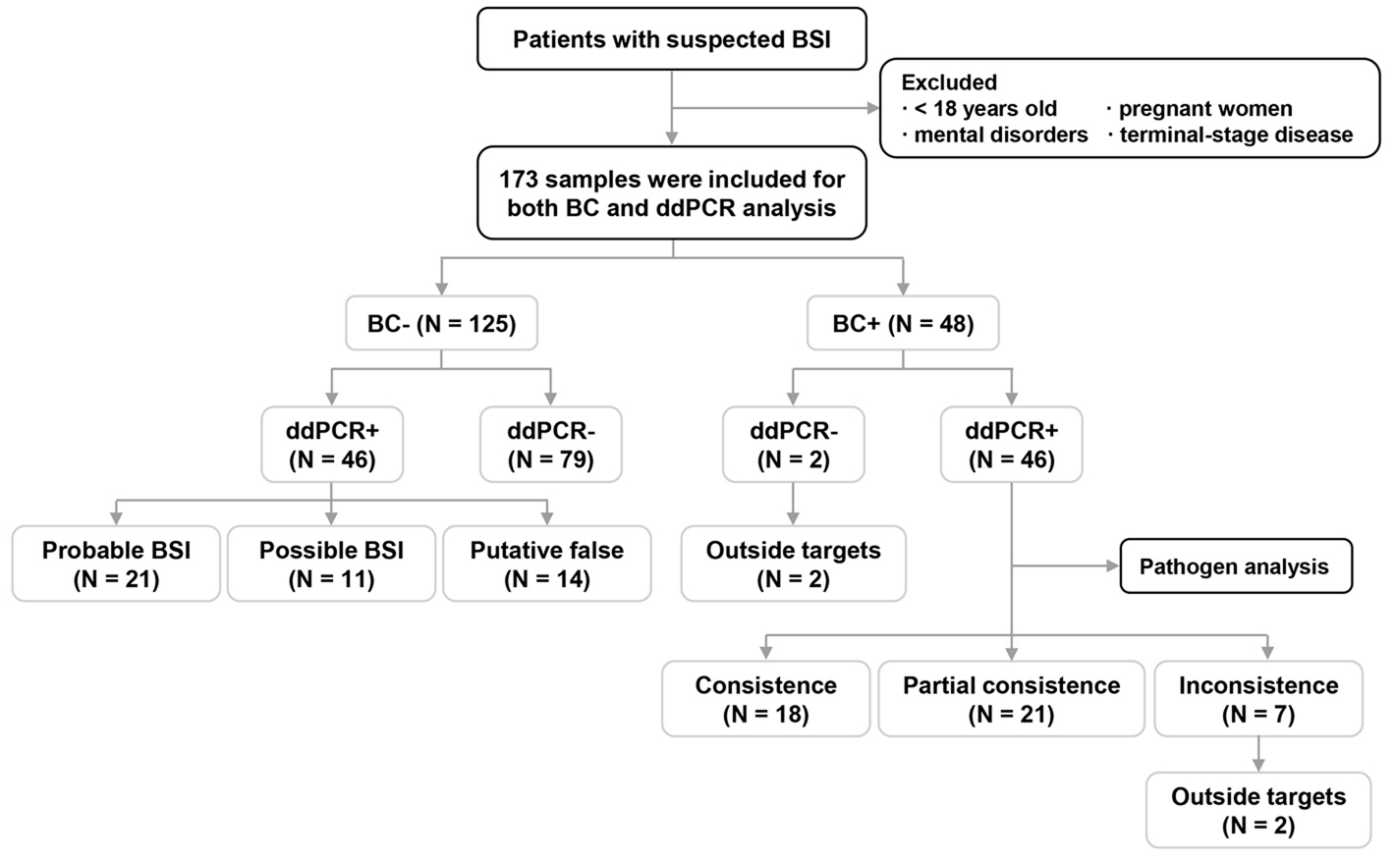
Flow chart for patient enrollment and overall analyses of blood culture (BC)/droplet digital PCR (ddPCR) results. BSI: bloodstream infection; +, samples with pathogen(s) detected; -, samples without a pathogen detected.

In this prospective study, 173 patients suspected of having BSIs were enrolled from October 2022 to October 2023 (**Figure 1**). The diagnosis of suspected BSI was made through the clinical judgment of the treating physicians according to systemic inflammatory response syndrome/sepsis criteria (Evans et al., 2021). The exclusion criteria included age < 18 years, having a mental disorder, being pregnant, or having any terminal-stage disease. Upon the suspicion of a BSI, whole blood samples were obtained for the BC and ddPCR assay simultaneously. Clinical and laboratory data were also collected.

This study was approved by the institutional review board of the First Affiliated Hospital of Sun Yat-sen University. Written informed consent was obtained from all patients or their legal representatives. All data were anonymized prior to analysis.

### 2.2 BC procedure

For each patient with a suspected BSI, at least two sets of BCs were collected according to routine clinical practice (BacT/ALERT^®^ VIRTUO System, bioMérieux, France) (Murray and Masur, 2012). Once the system showed a positive signal, gram staining and subculture were performed. Following overnight incubation, the pathogens were identified by matrix-assisted laser desorption/ionization time-of-flight mass spectrometry (VITEK^®^ MS system, bioMérieux, France). AST was performed using the VITEK^®^ system (bioMérieux, France). Interpretation of the AST results was based on the Clinical and Laboratory Standards Institute guidelines (CLSI, 2022). For *Enterobacterales* with a carbapenem-resistant phenotype, carbapenem-encoding resistance genes (*bla*_KPC_, *bla*_NDM_, *bla*_VIM_, *bla*_IMP_, and *bla*_OXA48_) were detected using a Xpert Carba-R Assay (Cepheid, USA) following the manufacturer’s instructions (Jin et al., 2021b). For most of *Staphylococcus* spp., cefoxitin was used to predict results for *mecA*-mediated methicillin (oxacillin) resistance (CLSI, 2022). If gram-positive (G^+^) strains were shown to be the vancomycin-resistant phenotype by AST, PCRs were performed to detect vancomycin-resistant genes as described previously (Selim, 2022).

### 2.3 ddPCR testing

For ddPCR testing, at least 5 mL of whole blood using EDTA anticoagulant was collected. After centrifuging at 1200×g for 5 min, 2 mL of plasma was collected for DNA extraction using the Easy-CF2 Nucleic Acid Extraction/Purification Kit (Pilot Gene Technologies, Hangzhou, China) according to the manufacturer’s instructions.

Further, ddPCR testing was conducted as previously described with some modifications via a multiplex ddPCR testing platform for research use only (Pilot Gene Technologies, Hangzhou, China) (Wu et al., 2022). Briefly, the reaction mixture containing 15 μL of DNA extract was passed through a micro-channel (Droplet Generator DG32) to generate tens of thousands of water-in-oil emulsion droplets. After PCR amplification using a Thermal Cycler TC1, droplet counts and amplitudes were scanned and analyzed by a CS7 chip scanner and Gene PMS software (V1.1.8.20221121). Copies of the targeted pathogens or genes were reported. The synthesized DNA fragment at a concentration of 10^4^ copies/mL was used as the positive control, which was prepared by inserting pathogen target DNA into the pUC57 plasmid. DNase-free water was used as the negative control. The testing process took < 2.5h in total.

**Table 1** presents the detection of pathogens and antimicrobial resistance (AMR) genes with the corresponding fluorescence channels. Considering the global and local pathogen epidemiological BC data (Jin et al., 2021a; Chen et al., 2023; Zhang et al., 2024), target pathogens included 12 common BSI bacteria, three common BSI fungi, and five main resistance markers. In addition, cases of clinical diagnosis of aspergillosis, mucormycosis, and *Pneumocystis jirovecii* pneumonia assisted by blood-based next-generation sequencing have been increasing in recent years (Cheng and Yu, 2022; Hoenigl et al., 2023; Wang et al., 2024), so the corresponding pathogens were also recommended for detection by clinicians in this study (**Table 1**).

**Table 1:** Pathogens and their corresponding fluorescence.

Panel	Fluorescence channel					
	FAM	VIC	ROX	CY5	CY5.5	A425
1	*Pseudomonas aeruginosa*	*Escherichia coli*	*Klebsiella pneumoniae*	*Acinetobacter baumannii*	/^a^	IC^b^
2	*Staphylococcus aureus*	*Candida* spp.	*Enterococcus* spp.	*Streptococcus* spp.	/	IC
3	*Stenotrophomonas maltophilia*	*Enterobacter cloacae*	*Proteus mirabilis*	Coagulase-negative staphylococci	*Serratia marcescens*	IC
4	*Pneumocystis jirovecii*	*Mucor & Rhizomucor* spp.	*Aspergillus* spp.	*Cryptococcus* spp.	*Talaromyces marneffei*	IC
5	*bla*_KPC_	*mecA*	*bla*_OXA48_	*bla*_NDM_ *+ bla*_IMP_	*VanA + VanM*	IC

^a^none; ^b^Internal control.

The detection sensitivity was determined according to the limit of detection (LoD) which was defined as the lowest amount of a target that is consistently detectable in at least 95% of the samples tested (Canchola et al., 2019). In this platform, LoD was determined by a series of the manufacturer’s experiments, of which two-fold serial dilutions of microbial DNA were taken. For each dilution of a pathogen, the lowest concentration was used as the LoD of the pathogen when at least 19 of 20 replicates could be detected. According to the manufacturer’s instructions, the detection sensitivity of this platform was 50 copies/mL for coagulase-negative *Staphylococci* (CoNS), *Streptococcus* spp., and *Candida* spp., and 25 copies/mL for the other microorganisms.

### 2.4 Definition and interpretation of the BSI and ddPCR results

The results of the ddPCRs and BCs were compared among the 173 samples. A positive BC (BC+) indicated a positive result in the BC, which excluded the possibility of contamination by clinical assessment. A positive ddPCR result (ddPCR+) indicated the presence of at least one target pathogen in ddPCR testing, while a negative result (ddPCR-) indicated the absence of any target pathogen. For ddPCR+/BC+ samples, pathogen results were further compared between the two methods. If the pathogen(s) via ddPCR testing displayed 100% or 0% concordance with BC, consistency or inconsistency was considered respectively; otherwise, partial consistency was considered. In addition, laboratory turnaround time (LTAT) refers to the time from receipt of the sample in the laboratory to the reporting of results (Of and For, 2014).

The results of the ddPCR were available to clinicians in real-time. Clinical infection and the outcome of these patients were verified by two trained physicians independently. A culture-proven BSI was defined as a patient’s samples being BC+ with systemic signs of infection, which may be secondary to a documented source or primary, according to the definitions released by the National Healthcare Safety Network (Timsit et al., 2020). If infection was suspected, all microbiological examinations were collected within 7 days of enrolment according to the standard microbiology laboratory procedures, which included routine microbiological cultures, microscopic examination, and other examinations such as metagenomic next-generation sequencing. A composite clinical infection standard was defined, consisting of all microbiological results and clinical adjudication (Kalligeros et al., 2020; Nguyen et al., 2019).

For ddPCR+/BC- results, the following classifications were used according to previous studies (Kalligeros et al., 2020; Nguyen et al., 2019): (i) probable: ddPCR result was concordant with a microbiological test performed within 7 days of sample collection from another extra-blood site; (ii) possible: without microbiological data but the ddPCR result had the potential for pathogenicity based on clinical presentation and laboratory findings; (iii) putative false: ddPCR result was inconsistent with clinical presentation.

### 2.5 Statistical analysis

Statistical analysis was performed using SAS 9.4, using Chi-squared test, analysis of variance, Student’s t-test, Wilcoxon Rank-Sum test, or logistic regression analysis, where applicable.

In addition, receiver operating characteristic (ROC) curves were constructed, and the areas under the ROC curves (AUCs) were estimated to determine the feasibility of using the DNA load of a pathogen as a predictor for a corresponding BSI. Furthermore, Cohen’s kappa coefficient of agreement was calculated, which was used to classify the level of concordance: poor (<0.00), slight (0.00-0.20), fair (0.21-0.40), moderate (0.41-0.60), substantial (0.61-0.80), almost perfect (0.81-1.00) (Landis and Koch, 1977).

A *P* value of < 0.05 was considered statistically significant.

## 3 Results

### 3.1 Patient characteristics

A total of 173 samples were obtained from the 173 patients for both BC and ddPCR testing. Patient characteristics are presented in **Table 2**. The average age of the patients was 63.9 years (range: 18 - 102), and 64.7% (112/173) were men. In total, 65.9% (114/173) of the patients were in the ICU.

**Table 2:** Patient characteristics and laboratory data of patients with suspected bloodstream infections.

Patient characteristic	BC+ and/or ddPCR+^a^ (n = 94)	BC-/ddPCR-^b^ (n = 79)	*P*
Age (years)	62.5 ± 16.3	65.6 ± 17.2	0.223
Male, n (%)	60 (63.8)	52 (65.8)	0.785
Intensive Care Unit, n (%)	60 (63.8)	54 (68.4)	0.785
Temperature (°C)	38.2 ± 1.1	37.9 ± 1.0	0.056
Laboratory data, median (IQR)			
White blood cell (× 10^9^/L)^c^	11.3 (6.5, 14.5)	11.4 (7.7, 16.2)	0.218
Neutrophil (%)^c^	87.3 (81.5, 93.1)	85.4 (79.4, 91.0)	0.141
Procalcitonin (ng/mL)	2.4 (0.6, 9.0)	0.68 (0.3, 2.5)	0.001
C-reactive protein (mg/L)	93.6 (56.9, 209.6)	107.3 (43.0, 145.8)	0.657
Clinical status, n (%)			
Cancer	34 (36.2)	26 (32.9)	0.654
Diabetes mellitus	37 (39.4)	37 (46.8)	0.322
Neutrophilic deficiency	8 (8.5)	6 (7.6)	0.826
Immunosuppression^d^	43 (45.7)	32 (40.5)	0.489
Pulmonary infection	51 (54.3)	58 (73.4)	0.009
Recent use of broad-spectrum antibiotics	67 (71.3)	67 (84.8)	0.034
28-day mortality, n (%)	19 (20.2)	18 (22.8)	0.681

^a^Patients with microorganisms detected by blood culture (BC) and/or droplet digital PCR (ddPCR) testing, including BC-/ddPCR+ (n = 46), BC+/ddPCR+ (n = 46), and BC+/ddPCR- (n = 2); ^b^patients without microorganisms detected by either method; ^c^data excluded patients with a neutrophilic deficiency; ^d^Immunosuppressive patients included those with malignancies, transplantation, or autoimmune conditions receiving immunosuppressant therapy.

As shown in **Figure 1**, microorganisms were detected in 94 samples (54.3%) by BC and/or ddPCR testing; of these, 92 were ddPCR+. Compared to BC-/ddPCR- patients, BC+ and/or ddPCR+ patients showed significantly higher levels of procalcitonin (PCT) (*P* = 0.001), while the prevalence of pulmonary infection (*P* = 0.009), and recent use of broad-spectrum antibiotics (*P* = 0.034) were significantly lower (**Table 2**).

### 3.2 Overall analysis of BC and ddPCR results

The results of BC and ddPCR were concordant in 125 samples, including 79 BC-/ddPCR- and 46 BC+/ddPCR+ samples (**Figure 1**). For the 46 BC+/ddPCR+ samples, the pathogen results were completely consistent in 18 samples, partially consistent in 21 samples, and inconsistent in 7 samples (**Figure 1**). When taking the pathogen results into consideration, the rate of samples with completely consistent results was 56.1% (97/173). The inconsistent results of seven BC+/ddPCR+ samples are listed in **Supplementary Table S1**.

**Figure 2:**
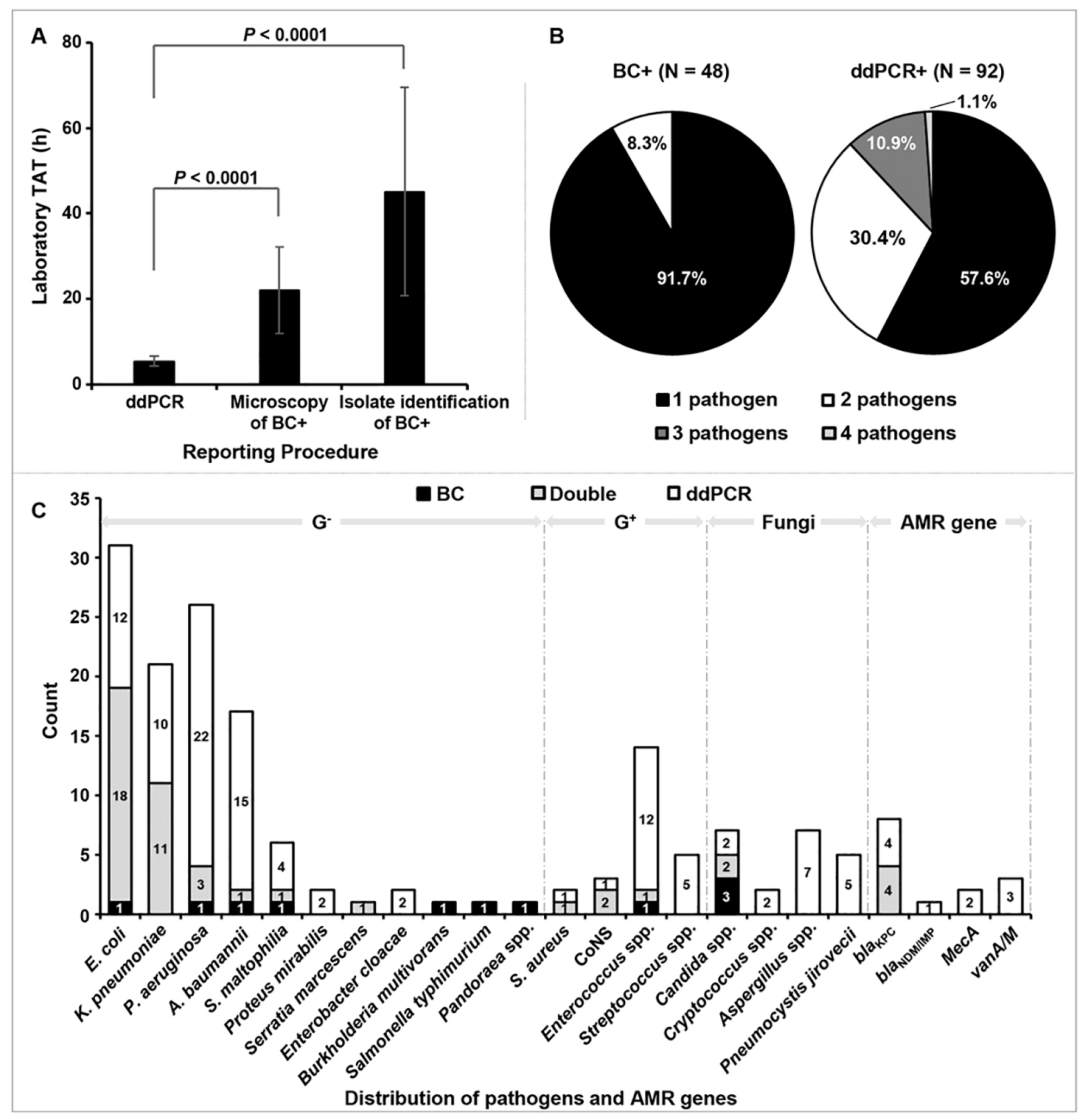
The comparison analysis between blood culture (BC) and droplet digital PCR (ddPCR) results. **(A)** Comparison analysis of laboratory TAT between BCs and ddPCRs in BC+/ddPCR+ samples except those with inconsistent results. Laboratory TAT: the time from receipt of the sample in the laboratory to the reporting of results. **(B)** Counts and percentages of co-infections in patients with positive BC (BC+) and positive ddPCR (ddPCR+) results. **(C)** Distribution characteristics of pathogens and antimicrobial resistance (AMR) genes detected by BC and ddPCR testing. G^-^/^+^: gram-negative/positive bacteria; CoNS (coagulase-negative *Staphylococci*) included *S. epidermidis* (n = 1) and *S. lugdunensis* (n = 1); *Enterococcus* spp. included *E*. *faecalis* (n = 1) and *E*. *faecium* (n = 1); *Candida* spp. included *C*. *tropicalis* (n = 2), and one of *C*. *albicans*, *C*. *parapsilosis*, and *C*. *auris* respectively.

For the 21 samples with partially concordant results, all the culture-proven pathogens were detected by ddPCR simultaneously. The LTAT was further analyzed among the BC+/ddPCR+ samples, excluding those with inconsistent results (n = 39) (**Figure 2A**). For the BCs, the average reporting time of microscopy and isolate identification was 22.0 h (6.9 - 44.2 h) and 45.1 h (17.4 - 103.0 h) respectively, much longer than that of ddPCR testing (5.4 h, 2 -7.8 h) (*P* < 0.0001).

In addition, there were two BC+/ddPCR- samples due to the microorganisms being outside the detection range of ddPCR (**Supplementary Table S1**). Of the 46 BC-/ddPCR+ samples combining microbiological and clinical evidence, 21 (45.7%) met the criteria for a probable BSI, 11 (23.9%) for a possible BSI, and the remaining 14 cases (30.4%) were putatively false (**Figure 1**).

**Table 3** lists the overall agreement between the BC and ddPCR results. The aggregate sensitivity, specificity, positive predictive value (PPV), and negative predictive value (NPV) for ddPCR were 81.3%, 63.2%, 45.9%, and 89.8%, respectively. Compared to G^+^ (80.0%) and G^-^ bacteria (87.2%), the sensitivity of ddPCR was much lower for fungi (40.0%). If both probable and possible BSIs were assumed to be positive, the anticipated sensitivity, specificity, PPV, and NPV of ddPCR for complicated BSIs were 88.8%, 86.0%, 84.5%, and 89.9%, respectively.

**Table 3:** The aggregate agreement of blood culture (BC) *vs.* droplet digital PCR (ddPCR).

Sample (n = 173)		ddPCR		Sensitivity (%)	Specificity (%)	PPV (%)	NPV (%)
		+	-				
Total	BC+	39	9^a^	81.3	63.2	45.9	89.8
	BC-	46	79				
G^-^	BC+	34	5^a^	87.2	70.9	46.6	95.0
	BC-	39	95				
G^+^	BC+	4	1^a^	80.0	88.7	17.4	99.3
	BC-	19	149				
Fungi	BC+	2	3^a^	40.0	91.1	11.8	98.1
	BC-	15	153				
Positive by clinical diagnosis		71	9	88.8	86.0	84.5	89.9
Negative by clinical diagnosis		13	80				

a Cases with completely inconsistent pathogens were considered as BC+/ddPCR-; PPV, positive predictive value; NPV, negative predictive value; G^-^, gram-negative bacteria; G^+^, gram-positive bacteria.

### 3.3 Pathogen and AMR gene analysis of BC results

Culture-proven BSIs were positive for 52 pathogens in 48 (27.7%) cases; a polymicrobial BSI was detected in 8.3% of cases (4/48) (**Figures 1**, **2B**). Among these pathogens, 42 (80.8%) were G^-^ bacteria; the three top strains were *Escherichia coli* (36.5%, 19/52), *Klebsiella pneumoniae* (21.2%, 11/52) and *Pseudomonas aeruginosa* (7.7%, 4/52) (**Figure 2C**). As shown in **Figure 2C**, 18 types of pathogens were detected by BC, four of which were outside the detection range of ddPCR, including *Burkholderia multivorans* (n = 1), *Salmonella typhimurium* (n = 1), *Pandoraea* spp. (n = 1), and *C. auris* (n = 1).

Additionally, four *bla*_KPC_ genes were found in carbapenem-resistant *K. pneumoniae* strains, which were detected by ddPCR simultaneously (**Figure 2C**). None of the carbapenem-resistant *Enterobacterales* strains expressed AMR genes of *bla*_NDM_, *bla*_VIM_, *bla*_IMP_, or *bla*_OXA48_. For *Staphylococcus* spp., none were methicillin-resistant. None of the G^+^ strains were vancomycin-resistant.

### 3.4 Pathogen and AMR gene analysis of the ddPCR testing

Among the 92 ddPCR+ cases, 53 (57.6%) had a single microorganism detected, while there were multiple microorganisms (2 - 4 pathogens) detected in the other 39 (42.4%) (**Figure 2B**). A total of 143 microorganisms were detected, comprising G^-^ strains (n = 102), G^+^ strains (n = 23), and fungal strains (n = 18); of them, 41 (28.7%) were detected by BC simultaneously. Within the detection range of ddPCR, 7 culture-proven pathogens were not found in the ddPCR testing (**Figure 2C**).

*E. coli* (21.0%, 30/143) was the most prevalent pathogen, followed by *P. aeruginosa* (17.5%, 25/143), *K. pneumoniae* (14.7%, 21/143), and *Acinetobacter baumannii* (11.2%, 16/143). All the carbapenem-resistant genes, *bla*_KPC_ (n = 8) and *bla*_NDM_ (n = 1), were detected from *K. pneumoniae* strains. For G^+^ microorganisms, *Enterococcus* spp. (9.1%, 13/143) was the most prevalent strain. Among the microorganisms from the BC-/ddPCR+ samples, the *vanA/vanM* genes were found in three strains of *Enterococcus* spp., and one CoNS strain was positive for the *MecA* gene in ddPCR testing. Besides *Candida* spp. (n = 4), the remaining 14 strains of fungi were *Aspergillus* spp. (n = 7), *Pneumocystis jirovecii* (n = 5), and *Cryptococcus* spp. (n = 2), respectively.

### 3.5 Association between pathogen loads by ddPCR testing and culture-proven BSIs

**Figure 3:**
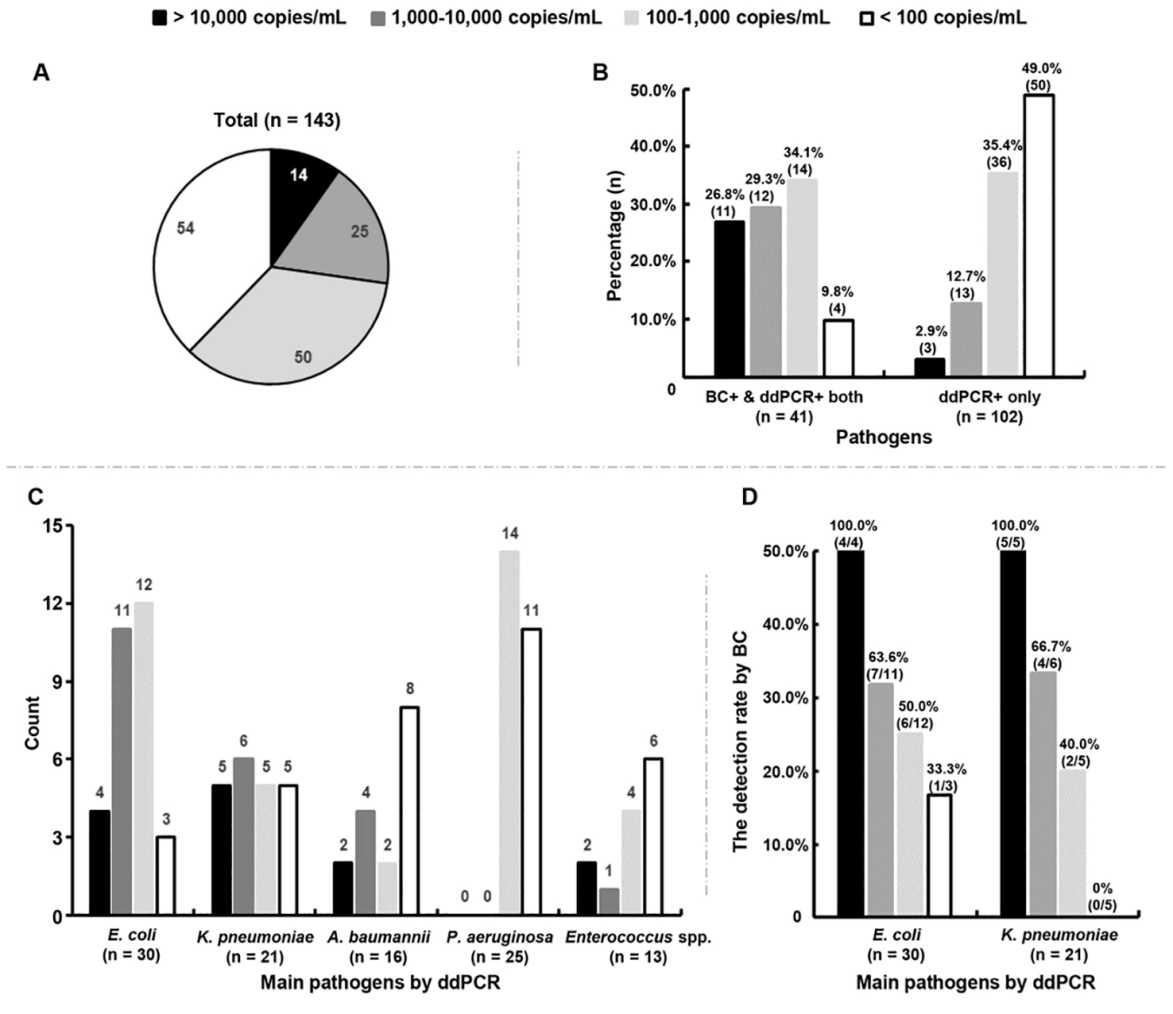
Association between positive blood cultures (BC+) and DNA loads measured by droplet digital PCR (ddPCR). **(A)** Distribution characteristics of the DNA loads of all microorganisms detected by ddPCR testing. **(B)** Percentages of microorganisms with different DNA loads in BC+/ddPCR+ and BC-/ddPCR+ groups, respectively. BC+/ddPCR+: microorganisms found both by BC and ddPCR; BC-/ddPCR+: microorganisms found by ddPCR only. **(C)** Count of main microorganisms with different DNA loads detected by ddPCR. **(D)** The rates of positive BCs of *E*. *coli* and *K*. *pneumoniae* strains with different DNA loads respectively.

The DNA loads of the microorganisms detected by ddPCR were further analyzed, showing a median level of 158.0 (range: 30.0 - 3.2 × 10^5^) copies/mL. In total, 72.7% (104/143) of the microorganisms had DNA loads < 1,000 copies/mL (**Figure 3A**). Among the 41 microorganisms detected by both methods, the rate of microorganisms with low levels (< 1,000 copies/mL) was 43.9% (18/41), much lower than the rate of those detected by ddPCR only (84.4%, 86/102) (*P* < 0.0001) (**Figure 3B**).

Of the top five microorganisms detected by ddPCR, 50.0% (15/30) of *E. coli* and 52.4% (11/21) of *K. pneumoniae* were found to have levels > 1,000 copies/mL, which were more than *A. baumannii* (37.5%, 6/16) and *Enterococcus* spp. (23.1%, 3/13) (**Figure 3C**). The detection rate of *E. coli* by BC was found to increase with increasing DNA loads (odds ratio = 1.002, 95% confidence interval = 1.001-1.002, *P* = 0.0002) (**Figure 3D**). A similar trend was also found for *K. pneumoniae* (odds ratio = 1.001, 95% confidence interval = 1.000-1.002, *P* = 0.037) (**Figure 3D**). Surprisingly, the DNA loads of *P. aeruginosa* were all below 500 copies/mL, 44.0% (11/25) of which were < 100 copies/mL (**Figure 3C**).

Among the two *A. baumannii* strains found by BC, only one had detected by ddPCR simultaneously with a DNA load of 49,427.0 copies/mL. *Enterococcus* spp. was detected in one sample by both methods with a DNA load of 301.5 copies/mL. The detection rate of *Staphylococcus aureus* by ddPCR was only 1.4% (2/143), with extremely low levels (< 35 copies/mL).

### 3.6 Evaluation of pathogen load as a potential BSI marker

**Figure 4:**
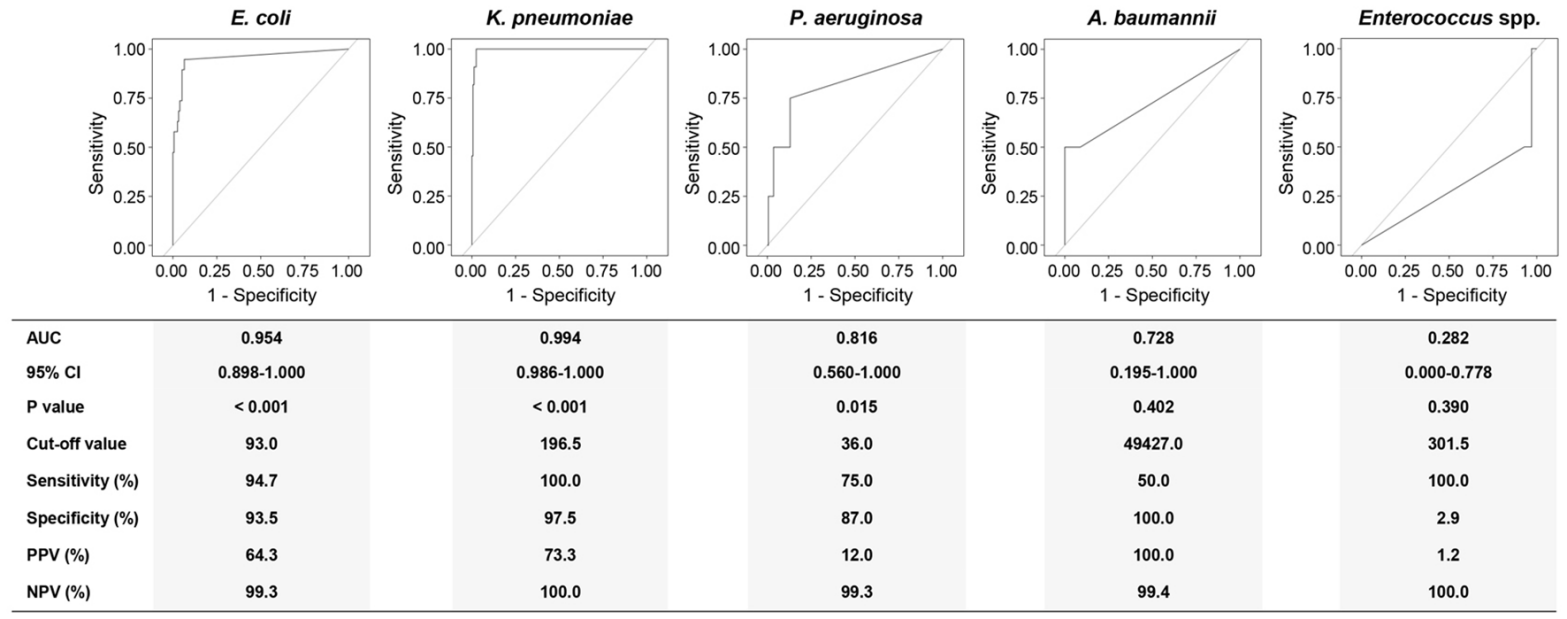
Receiver operating characteristics (ROC) curve analyses using the DNA loads of different pathogens for discriminating corresponding BSIs from suspected BSIs. AUC, area under curve; 95% CI, 95% confidence interval; PPV, positive predictive value; NPV, negative predictive value; Eco, *E. coli*; Kpn, *K. pneumoniae*; Pae, *P. aeruginosa*; Aba, *A. baumannii*; Ent, *Enterococcus* spp. Diagnostic sensitivity, specificity, PPV, and NPV were calculated at a cut-off point that maximized the value of the Youden index.

Taking BC as the gold standard, the ROC curve was used to reveal whether the pathogen load could be a potential marker for a corresponding BSI for the top five detected microorganisms by ddPCR (**Figure 4**).

The DNA load of *E. coli* discriminated an *E. coli* BSI from a suspected BSI with an AUC of 0.954 (95% CI: 0.898-1.000). At the cut-off value of 93.0 copies/mL, the sensitivity and specificity for *E. coli* levels were 94.7% and 93.3%, respectively. For *K. pneumoniae* BSI, the DNA load of *K. pneumoniae* at the cut-off value of 196.5 copies/mL yielded an AUC of 0.994 (95% CI: 0.986-1.000) with 100.0% sensitivity and 97.5% specificity. The cut-off value for *P. aeruginosa* was 36.0 copies/mL with 75.0% sensitivity and 87.0% specificity (AUC: 0.816, 95% CI: 0.560-1.000). Though the AUC was recorded as 0.728, the 95% CI for *A. baumannii* was 0.195-1.000 (*P* = 0.402). Remarkably, the AUC for *Enterococcus* spp. was 0.282 (95% CI: 0.000-0.778).

According to the ROC analyses above, we further performed a consistency analysis of the BC and ddPCR results (**Table 4**). The Kappa coefficient showed almost perfect and good agreement for *K. pneumoniae* (κ = 0.834) and *E. coli* (κ = 0.731) BSIs, respectively. Regrettably, there was a slight agreement for *P. aeruginosa* BSIs when comparing ddPCR to BC (κ = 0.167).

**Table 4:** Comparison of blood culture (BC) and droplet digital PCR (ddPCR) testing in terms of pathogen identification.

Pathogen identification	ddPCR rejudged by the cut-off point according to ROC analyses	BC		Cohen’s Kappa coefficient	*P* value
		+	-		
*Escherichia coli*	+	18	10	0.731	<0.001
	–	1	144		
*Klebsiella pneumoniae*	+	11	4	0.834	<0.001
	–	0	158		
*Pseudomonas aeruginosa*	+	3	23	0.167	0.001
	–	1	146		

ROC, receiver operating characteristic.

### 3.7 Clinical evaluation of ddPCR results

According to their clinical presentation and all the laboratory findings, the antibiotic treatment regimen was adjusted for a patient if a composite clinical infection was diagnosed by the treating physician.

*E. coli* and/or *K. pneumoniae* were detected in eight BC-/ddPCR+ samples with DNA loads above the corresponding cut-off values. For other samples that were BC-/ddPCR+, pathogens with DNA loads of > 1,000 copies/mL were detected in seven, including *A. baumannii* (n = 3), *Enterococcus* spp. (n = 2), and *Pneumocystis jirovecii* (n = 2). As presented in **Supplementary Table S2**, for these aforementioned BC-/ddPCR+ patients, the infection symptoms obviously improved when they received correspondingly adjusted antibiotic treatments.

## 4 Discussion

Though a series of studies have presented ddPCR as a promising tool for early pathogen diagnosis of BSIs and sepsis (Merino et al., 2022), more data are needed to support wider clinical application. In this study, we carefully evaluated the results and clinical impact of ddPCR in comparison with conventional BC and a clinical diagnosis.

In a clinical laboratory, a BC usually takes 6 h to 5 days for a pathogen to grow to detectable levels with additional time for its identification. Similar to Tabak’s findings (Tabak et al., 2018), the average reporting time of microscopic examination and isolate identification for BC in this study were 22.0 h (range: 6.9 - 44.2 h) and 45.1 h (range: 17.4 - 103.0 h), respectively. Excitingly, the LTAT can be substantially abridged by ddPCR without the need for culturing. The average LTAT here was shortened to 5.4 h (range: 2.0 h - 7.8 h) when using ddPCR, which was similar to others’ findings (Yin et al., 2024; Liu et al., 2023).

Additionally, ddPCR has an extremely high detection sensitivity, meaning it can detect additional cases that BC cannot (Shin et al., 2021). A series of studies showed the detection rates of ddPCR+ cases were 2.4 to 6.8-fold higher than those determined via BC (Li et al., 2024; Lin et al., 2023; Liu et al., 2023; Yin et al., 2024). A similar phenomenon was also observed here (ddPCR *vs.* BC: 53.2% *vs.* 27.7%). Lin et al. found that the detection rate of ddPCR was positively correlated with several laboratory results, such as PCT (Lin et al., 2023). In this study, patients who were BC+ and/or ddPCR+ showed significantly higher PCT levels with lower rates of pulmonary infection and recent use of broad-spectrum antibiotics when compared to BC-/ddPCR- patients (**Table 2**). Considering serum PCT increases along with the severity of bacterial infection (Gregoriano et al., 2020), this suggested that these positive patients might suffer much more severe infections. For BC-/ddPCR- patients, we speculated a local infection might be the main cause of the symptoms without pathogens being released into the blood.

The ddPCR testing also exhibited a much higher detection rate of mixed pathogens in comparison with BC, which might be associated with poor prognosis (Li et al., 2023; Liu et al., 2023; Wu et al., 2022; Zhao et al., 2024). Of the 92 ddPCR+ cases here, multiple microorganisms were detected in 39 (42.4%)(**Figure 2B**). Remarkably, patients with multiple microorganisms presented with higher PCT levels (*P* = 0.002) and temperatures (*P* = 0.001) while there was a lower percentage of pulmonary infection (*P* = 0.009) (**Supplementary Table S3**). However, it is difficult to conclude whether these mixed pathogens were all truly agents of a BSI or not, since microbial cell-free DNA detected by ddPCR in the plasma can still be detected almost 2 weeks after BC becomes negative (Eichenberger et al., 2022). Further exploration is needed to elucidate whether ddPCR truly provides a higher detection rate of mixed infections.

Consistent with the high detection rates above, the total number of microorganisms detected by ddPCR was much higher than that by BC (Li et al., 2024; Wu et al., 2022). However, ddPCR did not have much better sensitivity and specificity compared with the BC results. For BC-validated BSIs, ddPCR had a median sensitivity of 75.0% (range: 72.5% - 90.0%), and an aggregate specificity ranging from 51.0% to 75.4% (Li et al., 2023; Li et al., 2024; Lin et al., 2023; Wu et al., 2022). Here ddPCR detected 143 microorganisms, displaying an aggregate sensitivity and specificity of 81.3% and 63.2%, respectively. When clinically diagnosed BSI criteria were used as a comparison, the sensitivity and specificity of ddPCR increased, ranging from 78.1% to 92.6% and 78.5% to 92.5%, respectively (Li et al., 2023; Li et al., 2024; Wu et al., 2022). When considering clinically validated BSIs, we also observed that the value of ddPCR improved with a sensitivity of 88.8% and a specificity of 86.0%. Nevertheless, the true value of ddPCR might be ambiguous when considering the overall analysis.

For the first time, we have investigated the correlation between the DNA load of a pathogen detected by ddPCR and its corresponding BC result. Similar to the prevalence of bloodstream isolates in China (Jin et al., 2021a), *E. coli* and *K. pneumoniae* were the most common BSI-causing pathogens here; for them, the positive rates of BC were both found to increase along with DNA loads (*P* < 0.05). Compared to the BC results, the sensitivity of ddPCR for *E. coli* and *K. pneumoniae* reached up to 94.7% and 100.0%, respectively. Most importantly, the AUCs for both pathogens were > 0.95 with good Kappa agreements, suggesting the DNA loads of both pathogens might be excellent predictors for corresponding culture-proven BSIs. For the BC-negative patients with the DNA loads of *E. coli* (> 93.0 copies/mL) and/or *K. pneumoniae* (> 196.5 copies/mL) above cut-off values, the infection symptoms obviously improved during adjusted antibiotic treatment (**Supplementary Table S2**).

The detection rate of *P. aeruginosa* (7.7%, 4/52) was found to be lower than that of *K. pneumoniae* (21.2%, 11/52) among the 52 culture-proven pathogens; however, the results were reversed in the ddPCR testing (count: 25 *vs.* 21). Interestingly, the DNA loads of *P. aeruginosa* by ddPCR were all below 500 copies/mL, 44.0% (11/25) of which were below 100 copies/mL, which might be associated with the relatively low positivity rate of *P. aeruginosa* BSIs. Though the AUC was 0.816 (95% CI: 0.560-1.000), the slight Kappa agreement (κ = 0.167) hinted that the DNA load of *P. aeruginosa* was not suitable for predicting a corresponding culture-proven BSI.

In addition, the DNA loads of *A. baumannii* and *Enterococcus* spp. were also found to have little predictive value for corresponding BSIs. For *A. baumannii*, though the AUC was 0.728, the 95% CI (0.195-1.000) included 0.5, meaning the ROC curve was not statistically significant (*P* = 0.402). The AUC for *Enterococcus* spp. was 0.282 (95% CI: 0.000-0.778). Surprisingly, though 37.5% (6/16) of *A. baumannii* and 23.1% (3/13) of *Enterococcus* spp. had high DNA levels (> 1,000 copies/mL), only one *A. baumannii* case was detected by BC simultaneously. Two reasons might be associated with the failure to detect eight high-level pathogens in BC, including the existence of other culture-proven pathogens with higher DNA loads (n = 3), and the use of antibiotics prior to blood collection (n = 5, **Supplementary Table S2**). This hinted that the positivity rates of these pathogens might be grossly underestimated in BSIs according to BC results.

Several limitations nevertheless deserve mention. First, ddPCR has limited detection targets for clinical pathogens. Although target pathogens covered over 90% of the BSI pathogens in our lab, some culture-proven pathogens were not detected by ddPCR. Second, the reason for there being BC+/ddPCR- pathogens within the detection range might be associated with the detection performance of the ddPCR platform. Further optimization of the ddPCR reaction system and conditions is needed. Third, though we detected several major AMR genes and observed good consistency between the detection of the *bla*_KPC_ gene and AST results, the predictive value of bacterial resistance using AMR genes is still controversial and needs further verification due to the many reasons for bacterial resistance. Fourth, though ddPCR is thought to have high reproducibility (Pinheiro et al., 2012; Hu et al., 2021), more studies are still needed to evaluate its performance by comparing it to other molecular technologies. Finally, as we were limited by the sample size, our results warrant further investigations.

In conclusion, the performance of the multiplex ddPCR system in this study exhibited higher detection sensitivity and faster turnaround times when compared to BC. Notably, this study demonstrated that the DNA loads of *E. coli* and *K. pneumoniae* had excellent predictive value for corresponding BSIs, the cut-off values of which were 93.0 copies/mL and 196.5 copies/mL, respectively. Our findings suggest that ddPCR is a promising tool for early pathogen detection and timely targeted treatment for patients with *E. coli* and/or *K. pneumoniae* BSIs in clinical settings. Further studies are needed to explore the predictive potential of ddPCR for other pathogens.

## Data Availability

The original contributions presented in the study are included in the article/**Supplementary Material**. Further inquiries can be directed to the corresponding authors.

## References

[B1] Abram T. J. Cherukury H. Ou C. Y. Vu T. Toledano M. Li Y.. (2020). Rapid bacterial detection and antibiotic susceptibility testing in whole blood using one-step, high throughput blood digital PCR. Lab. Chip. 20, 477–489. doi: 10.1039/c9lc01212e 31872202 PMC7250044

[B2] Canchola J. A. Vaks J. E. Tang S. (2019). Limit of detection (LoD) estimation using maximum likelihood from (Hit) rate data: The LoD_MLE SAS Macro. Proceedings of the Western Users of SAS Software Annual Conference, September 2019, Seattle, WA. doi: 10.13140/RG.2.1.2964.4886

[B3] Chen M. Hu D. Li T. Zheng D. Liao W. Xia X.. (2023). The epidemiology and clinical characteristics of fungemia in a tertiary hospital in southern China: a 6-year retrospective study. Mycopathologia. 188, 353–360. doi: 10.1007/s11046-023-00757-7 37380875

[B4] Cheng M. P. Stenstrom R. Paquette K. Stabler S. N. Akhter M. Davidson A. C.. (2019). Blood culture results before and after antimicrobial administration in patients with severe manifestations of sepsis: a diagnostic study. Ann. Intern. Med. 171, 547–554. doi: 10.7326/M19-1696 31525774

[B5] Cheng Z. Yu F. (2022). Clinical value of metagenomic next-generation sequencing in immunocompromised patients with sepsis. Med. Sci. Monitor. 28, e937041. doi: 10.12659/MSM.937041 PMC9380443 35957507

[B6] CLSI (2022). “Performance standards for antimicrobial susceptibility testing,” in CLSI Standard M100, 32nd ed. Ed. Wayne P. A. (USA: clinical and laboratory standards institute).

[B7] Eichenberger E. M. de Vries C. R. Ruffin F. Sharma-Kuinkel B. Park L. Hong D.. (2022). Microbial cell-free DNA identifies etiology of bloodstream infections, persists longer than conventional blood cultures, and its duration of detection is associated with metastatic infection in patients with *Staphylococcus aureus* and gram-negative bacteremia. Clin. Infect. Dis. 74, 2020–2027. doi: 10.1093/cid/ciab742 34460909 PMC9187311

[B8] Evans L. Rhodes A. Alhazzani W. Antonelli M. Coopersmith C. M. French C.. (2021). Surviving sepsis campaign: international guidelines for management of sepsis and septic shock 2021. Crit. Care Med. 49, e1063–e1143. doi: 10.1097/CCM.0000000000005337 34605781

[B9] Gregoriano C. Heilmann E. Molitor A. Schuetz P. (2020). Role of procalcitonin use in the management of sepsis. J. Thorac. Dis. 12, S5–S15. doi: 10.21037/jtd.2019.11.63 32148921 PMC7024752

[B10] Hoenigl M. Egger M. Price J. Krause R. Prattes J. White P. L. (2023). Metagenomic next-generation sequencing of plasma for diagnosis of COVID-19-associated pulmonary aspergillosis. J. Clin. Microbiol. 61, e0185922. doi: 10.1128/jcm.01859-22 36809121 PMC10035327

[B11] Hu B. Tao Y. Shao Z. Zheng Y. Zhang R. Yang X.. (2021). A comparison of blood pathogen detection among droplet digital PCR, metagenomic next-generation sequencing, and blood culture in critically ill patients with suspected bloodstream infections. Front. Microbiol. 12. doi: 10.3389/fmicb.2021.641202 PMC8165239 34079528

[B12] Huggett J. F. Cowen S. Foy C. A. (2015). Considerations for digital PCR as an accurate molecular diagnostic tool. Clin. Chem. 61, 79–88. doi: 10.1373/clinchem.2014.221366 25338683

[B13] Jin X. Zhang H. Wu S. Qin X. Jia P. Tenover F. C.. (2021b). Multicenter evaluation of Xpert Carba-R assay for detection and identification of the carbapenemase genes in rectal swabs and clinical isolates. J. Mol. Diagn. 23, 111–119. doi: 10.1016/j.jmoldx.2020.10.017 33212263

[B14] Jin L. Zhao C. Li H. Wang R. Wang Q. Wang H. (2021a). Clinical profile, prognostic factors, and outcome prediction in hospitalized patients with bloodstream infection: results from a 10-year prospective multicenter study. Front. Med. 8. doi: 10.3389/fmed.2021.629671 PMC8172964 34095163

[B15] Kalligeros M. Zacharioudakis I. M. Tansarli G. S. Tori K. Shehadeh F. Mylonakis E. (2020). In-depth analysis of T2Bacteria positive results in patients with concurrent negative blood culture: a case series. BMC Infect. Dis. 20, 326. doi: 10.1186/s12879-020-05049-9 32380973 PMC7206677

[B16] Lamy B. Sundqvist M. Idelevich E. A. (2020). Bloodstream infections - standard and progress in pathogen diagnostics. Clin. Microbiol. Infect. 26, 142–150. doi: 10.1016/j.cmi.2019.11.017 31760113

[B17] Landis J. R. Koch G. G. (1977). The measurement of observer agreement for categorical data. Biometrics. 33, 159–174. doi: 10.2307/2529310 843571

[B18] Li N. Cai Q. Miao Q. Song Z. Fang Y. Hu B. (2021). High-throughput metagenomics for identification of pathogens in the clinical settings. Small Methods 5, 2000792. doi: 10.1002/smtd.202000792 33614906 PMC7883231

[B19] Li Y. Huang K. Yin J. Tan Z. Zhou M. Dai J.. (2024). Clinical evaluation of a multiplex droplet digital PCR for pathogen detection in critically ill COVID-19 patients with bloodstream infections. Infection. 52, 1027–1039. doi: 10.1007/s15010-023-02157-x 38127118 PMC11143000

[B20] Li M. Zhao L. Zhu Y. Ou M. Xu H. Hu X.. (2023). Clinical value of droplet digital PCR in the diagnosis and dynamic monitoring of suspected bacterial bloodstream infections. Clin. Chim. Acta 550, 117566. doi: 10.1016/j.cca.2023.117566 37776990

[B21] Lin K. Zhao Y. Xu B. Yu S. Fu Z. Zhang Y.. (2023). Clinical diagnostic performance of droplet digital PCR for suspected bloodstream infections. Microbiol. Spectr. 11, e0137822. doi: 10.1128/spectrum.01378-22 36602351 PMC9927361

[B22] Liu W. Wang C. Pan F. Shao J. Cui Y. Han D.. (2023). Clinical application of a multiplex droplet digital PCR in the rapid diagnosis of children with suspected bloodstream infections. Pathogens. 12, 719. doi: 10.3390/pathogens12050719 37242389 PMC10223642

[B23] Massart N. Wattecamps G. Moriconi M. Fillatre P. (2021). Attributable mortality of ICU acquired bloodstream infections: a propensity-score matched analysis. Eur. J. Clin. Microbiol. Infect. Dis. 40, 1673–1680. doi: 10.1007/s10096-021-04215-4 33694037 PMC7945601

[B24] Merino I. de la Fuente A. Dominguez-Gil M. Eiros J. M. Tedim A. P. Bermejo-Martin J. F. (2022). Digital PCR applications for the diagnosis and management of infection in critical care medicine. Crit. Care 26, 63. doi: 10.1186/s13054-022-03948-8 35313934 PMC8935253

[B25] Murray P. R. Masur H. (2012). Current approaches to the diagnosis of bacterial and fungal bloodstream infections in the intensive care unit. Crit. Care Med. 40, 3277–3282. doi: 10.1097/CCM.0b013e318270e771 23034460 PMC4201853

[B26] Nguyen M. H. Clancy C. J. Pasculle A. W. Pappas P. G. Alangaden G. Pankey G. A.. (2019). Performance of the T2Bacteria panel for diagnosing bloodstream infections: a diagnostic accuracy study. Ann. Intern. Med. 170, 845–852. doi: 10.7326/M18-2772 31083728

[B27] Of I. For B. C. (2014). UK standards for microbiology investigations: Investigation of blood cultures (for organisms other than Mycobacterium species). Transport. 1-14. Available online at: https://www.gov.uk/uk-standards-for-microbiology-investigations-smi-qualityand-consistency-in-clinical-laboratories.

[B28] Peri A. M. Harris P. Paterson D. L. (2022). Culture-independent detection systems for bloodstream infection. Clin. Microbiol. Infect. 28, 195–201. doi: 10.1016/j.cmi.2021.09.039 34687856

[B29] Pinheiro L. B. Coleman V. A. Hindson C. M. Herrmann J. Hindson B. J. Bhat S.. (2012). Evaluation of a droplet digital polymerase chain reaction format for DNA copy number quantification. Anal. Chem. 84, 1003–1011. doi: 10.1021/ac202578x 22122760 PMC3260738

[B30] Selim S. (2022). Mechanisms of gram-positive vancomycin resistance (Review). Biomed. Rep. 16, 7. doi: 10.3892/br.2021.1490 34938536 PMC8686198

[B31] Shin J. Shina S. Jung S. H. Park C. Cho S. Y. Lee D. G.. (2021). Duplex dPCR system for rapid identification of gram-negative pathogens in the blood of patients with bloodstream infection: a culture-independent approach. J. Microbiol. Biotechnol. 31, 1481–1489. doi: 10.4014/jmb.2103.03044 34528911 PMC9705831

[B32] Tabak Y. P. Vankeepuram L. Ye G. Jeffers K. Gupta V. Murray P. R. (2018). Blood culture turnaround time in U.S. acute care hospitals and implications for laboratory process optimization. J. Clin. Microbiol. 56, e00500-e00518. doi: 10.1128/JCM.00500-18 30135230 PMC6258864

[B33] Timsit J. F. Ruppe E. Barbier F. Tabah A. Bassetti M. (2020). Bloodstream infections in critically ill patients: an expert statement. Intensive Care Med. 46, 266–284. doi: 10.1007/s00134-020-05950-6 32047941 PMC7223992

[B34] Wang W. Yao Y. Li X. Zhang S. Zeng Z. Zhou H.. (2024). Clinical impact of metagenomic next-generation sequencing of peripheral blood for the diagnosis of invasive mucormycosis: a single-center retrospective study. Microbiol. Spectr. 12, e0355323. doi: 10.1128/spectrum.03553-23 38095467 PMC10782995

[B35] Wu J. Tang B. Qiu Y. Tan R. Liu J. Xia J.. (2022). Clinical validation of a multiplex droplet digital PCR for diagnosing suspected bloodstream infections in ICU practice: a promising diagnostic tool. Crit. Care 26, 243. doi: 10.1186/s13054-022-04116-8 35941654 PMC9358819

[B36] Yin S. Lin Y. Wang B. Peng Y. Wang Z. Zhu X.. (2024). Reliability of droplet digital PCR alone and in combination with interleukin-6 and procalcitonin for prognosis of bloodstream infection. Infect. Drug Resistance. 17, 1051–1071. doi: 10.2147/IDR.S439683 PMC10950090 38505247

[B37] Zhang C. Fu X. Liu Y. Zhao H. Wang G. (2024). Burden of infectious diseases and bacterial antimicrobial resistance in China: a systematic analysis for the global burden of disease study 2019. Lancet Reg. Health-W. Pac. 43, 100972. doi: 10.1016/j.lanwpc.2023.100972 PMC10700598 38076321

[B38] Zhao Y. Lin K. Zhang H. Zhang Y. Li S. Zhang S.. (2024). Prognostic value of poly-microorganisms detected by droplet digital PCR and pathogen load kinetics in sepsis patients: a multi-center prospective cohort study. Microbiol. Spectr. 12, e0255823. doi: 10.1128/spectrum.02558-23 38526296 PMC11064489

[B39] Zheng Y. Jin J. Shao Z. Liu J. Zhang R. Sun R.. (2021). Development and clinical validation of a droplet digital PCR assay for detecting *Acinetobacter baumannii* and *Klebsiella pneumoniae* in patients with suspected bloodstream infections. Microbiologyopen. 10, e1247. doi: 10.1002/mbo3.1247 34964298 PMC8594765

