# Supplementary material for: Clinical evaluation of a multiplex droplet digital PCR for diagnosing suspected bloodstream infections: a prospective study
Source: Front Cell Infect Microbiol. 2025 Jan 16;14:1489792. doi: 10.3389/fcimb.2024.1489792 (PMC11779721; doi:10.3389/fcimb.2024.1489792)
Supplement: Supplementary file 1 [file Table1.docx]

**Table S1.** Pathogens distribution among positive blood culture (BC) samples with inconsistent droplet digital PCR (ddPCR) results.

| Cases | Pathogens | |
| --- | --- | --- |
|  | BC | ddPCR (DNA load, copies/mL) |
| 23 | *Pandoraea* spp. | *Negative* |
| 53 | *E. faecalis* | *Aspergillus* spp*.* (40.0) |
| 59 | *Salmonella typhimurium* | *Negative* |
| 62 | *C. albicans* | *A. baumannii* (56.0) |
| 130 | *C. parapsilosis* | *K. pneumoniae* (123.0)*, Enterococcus* spp*.* (99.5) |
| 141 | *C. auris* | *A. baumannii* (120.0)*, S. maltophilia* (31.0) |
| 151 | *Burkholderia multivorans* | *A. baumannii* (10782.5) |
| 171 | *E. coli* | *P. aeruginosa* (378.5) |
| 175 | *A. baumannii, S. maltophilia* | *Aspergillus* spp*.* (120.0) |

**Table S2.** Clinical therapy and outcome of some patients with negative blood culture (BC)/positive droplet digital PCR (ddPCR) results.

| Case | DNA load (copies/mL) | Extra-blood result^a^ | Therapy regimen according to clinical presentation, BC, ddPCR results, and other laboratory findings | | Anti-infection effect |
| --- | --- | --- | --- | --- | --- |
|  |  |  | Before ddPCR results | Adjusted after ddPCR results |  |
| 24 | Eco (2966.5) | None | Metronidazole, 0.5g QD IVDRIP | IPM, 0.5g Q6H IVDRIP + TGC, 0.05g Q12H IVDRIP | Improved |
| 27 | Eco (132.0) | None | SCF, 3g Q12H IVDRIP | IPM, 1g Q8H IVDRIP | Improved |
| 30 | Eco (1307.5) | None | None | SCF, 3g Q12H IVDRIP | Improved |
| 55 | Eco (219.5) | None | None | MEM, 1g Q12H IVDRIP | Improved |
| 63 | Eco (1124.0) | None | None | Piperacillin/tazobactam, 4.5g Q8H IVDRIP | Improved |
| 84 | Eco (1845.5) | None | MEM, 1g Q12H IVDRIP | MEM, 1g Q12H IVDRIP | Improved |
| 184 | Eco (260.0) | Drainage fluid | None | SCF, 3g Q8H IVDRIP | Improved |
|  | Kpn (7245.5) |  |  |  |  |
| 75 | Eco (573.0) Kpn (226.5) | None | None | IPM, 1g Q12H IVDRIP | Improved |
| 87 | Aba (5496.5) | None | TGC, 0.05g Q12H IVDRIP + MEM, 0.5g Q12H IVDRIP | PB, 500,000U Q12H IVDRIP + SCF, 3g Q6H IVDRIP | Improved |
| 94 | Aba (2862.5) | Sputum | TGC, 0.05g Q12H IVDRIP + MEM, 0.5g Q6H IVDRIP + Sulbactam, 1g Q6H IVDRIP | PB, 500,000U Q12H IVDRIP + TGC, 0.05g Q12H IVDRIP | Improved |
| 128 | Ent (14224) | None | CZA, 2.5g Q8H IVDRIP | VA, 0.5g Q12H IVDRIP (Initial dose: 1g) + CZA, 2.5g Q8H IVDRIP | Improved |
| 143 | Ent (10205) | Drainage fluid | IPM, 0.5g Q8H IVDRIP + SCF, 3g Q12H IVDRIP | VA, 0.5g Q12H IVDRIP (Initial dose: 1g) + IPM, 0.5g Q8H IVDRIP | Improved |
| 179 | Aba (3166.0) | None | IPM, 2g Q8H IVDRIP | TGC, 0.05g Q12H IVDRIP (Initial dose: 1g) | Improved |
| 25 | Pj (1325.5) | None | None | SXT, 0.96g Q6H nasal feeding + Levofloxacin, 0.5g QD IVDRIP | Improved |
| 58 | Pj (2639.0) | None | None | SXT, 0.96g Q12H PO +Moxifloxacin, 0.4g QD PO | Improved |

^a^: corresponding pathogen detected within 7 days from other extra-blood site (s); Eco: *E. coli*; Kpn: *K. pneumoniae*; Aba: *A. baumannii*; Ent: *Enterococcus* spp.; Pj: *pneumocystis jirovecii*; IPM: imipenem; TGC: tigecycline; SCF: cefoperazone/sulbactam; MEM: meropenem; PB: polymyxin B; CZA: ceftazidime/avibactam; VA: vancomycin; SXT, compound sulfamethoxazole tablets.

Table S3. Patient characteristics and laboratory data among patients with different droplet digital PCR (ddPCR) results.

| Patient characteristics | ddPCR-  (n = 81) | ddPCR+ with 1 pathogen (n = 53) | ddPCR+ with ≥ 2 pathogens (n = 39) | *P* |
| --- | --- | --- | --- | --- |
| Age (years) | 65.5 ± 17.1 | 64.7 ± 15.6 | 59.5 ± 17.1 | 0.261 |
| Male, n (%) | 54 (66.7) | 31 (58.5) | 27 (69.2) | 0.501 |
| Intensive Care Unit, n (%) | 56 (69.1) | 37 (69.8) | 21 (53.8) | 0.196 |
| Temperature (°C) | 37.9 ± 1.0 | 38.0 ± 1.0 | 38.7 ± 1.1 | 0.001 |
| Laboratory data, median (IQR) | | | | |
| White blood cell (× 10^9^/L)^a^ | 11.4 (7.7, 15.8) | 11.0 (6.9, 14.4) | 13.4 (6.8, 15.2) | 0.677 |
| Neutrophil (%)^a^ | 85.4 (79.2, 90.9) | 87.3 (80.7, 92.4) | 89.4 (82.9, 93.6) | 0.113 |
| Procalcitonin (ng/mL) | 0.7 (0.3, 3.3) | 1.4 (0.4, 4.1) | 3.2 (0.7, 16.0) | 0.002 |
| C-reactive protein (mg/L) | 108.2 (44.6, 146.3) | 89.0 (61.0, 198.6) | 96.0 (55.1, 219.1) | 0.932 |
| Clinical status, n (%) | | | | |
| Cancer | 26 (32.1) | 18 (34.0) | 16 (41.0) | 0.624 |
| Diabetes mellitus | 37 (45.7) | 20 (37.7) | 17 (43.6) | 0.657 |
| Neutrophilic deficiency | 6 (7.4) | 4 (7.5) | 4 (10.3) | 0.853 |
| Immunosuppression^b^ | 34 (42.0) | 22 (41.5) | 19 (48.7) | 0.743 |
| Pulmonary infection | 60 (74.1) | 31 (58.5) | 18 (46.2) | 0.009 |
| Recent use of broad-spectrum antibiotics | 68 (84.0) | 35 (66.0) | 31 (79.5) | 0.050 |
| 28-day mortality, n (%) | 18 (22.2) | 9 (17.0) | 10 (25.6) | 0.410 |

^a^: data excluded those from patients with neutrophilic deficiency; ^b^: Immunosuppressive patients included those with malignancies, transplantation, or autoimmune conditions under immunosuppressant therapy.
